# Dark Aberrant Crypt Foci with activated Wnt pathway are related to tumorigenesis in the colon of AOM-treated rat

**DOI:** 10.1186/1756-9966-27-26

**Published:** 2008-08-05

**Authors:** Qing Lu, Bo Jiang, Chen Lin, Tao Shan

**Affiliations:** 1 The Institute for Digestive Medicine, Nanfang Hospital, Southern Medical University, Guangzhou 510515, PR China; 2 Department of Gastroenterology, Guilin Medical College affiliated hospital, Guilin 541001, PR China

## Abstract

**Background:**

To evaluate the relationship between Aberrant Crypt Foci (ACF) and tumorigenesis, we observed the sequential development from ACF to tumor in the colon of azoxymethane-exposed wistar rats.

**Methods:**

Sixty wistar rats were sacrificed at different time points after exposure to azoxymethane and the colons were stained with methylene blue for stereomicroscopic analysis.

**Results:**

We found two types of early lesions: classic ACF and dark ACF. Dark ACF were characterized by dark blue staining, mildly enlarged or small compressed crypts that are not elevated from the surrounding epithelium. Large dark ACF and nascent tumors displayed the same surface morphology. Furthermore, dark ACF grew significantly faster than classic ACF and showed dysplasia without hyperplasia. In contrast, classic ACF showed hyperplasia without dysplasia. Dark ACF has significant higher expression rate of β-catenin (100%) and MMP-7 (81.82%) compared with the expression of β-catenin and MMP-7 in classic ACF (4.84% and 7.87%, respectively).

**Conclusion:**

Our data indicated that dark ACF is closely related to tumorigenesis while classic ACF is not. Furthermore, Wnt signal pathway was activated during the early period of dark ACF.

## Background

Aberrant crypt foci (ACF) were first described by Bird and Good. The lesion of ACF is composed of the enlarged crypts that are slightly elevated above the surrounding mucosa and the densely stained crypts with methylene blue [1]. ACF are considered as putative preneoplastic lesions of the colon in both humans and experimental animals [2,3].

Although ACF may share some morphologic, genetic, and biochemical features with colonic tumors [4-7], conflicting evidence has also been reported. For example, mutations in the K-*ras *gene are relatively common in human ACF, but are only detected at a relatively late stage of colon cancer [8-11]. In rat models, although hundreds of ACF are induced per animal by azoxymethane (AOM), and K-*ras *mutations are frequently observed in those ACF, only a few colon tumors are observed per animal [12-14]. In azoxymethane/dimethylhydrazine-treated rats and in patients with sporadic colorectal cancer, the number of tumors is minuscule compared with the large number of ACF [15], demonstrating that only a very small fraction of the ACF in theory has the potential to progress to the stage of a tumor. Therefore, there is a strong need to investigate the role of ACF in colon carcinogenesis. Alexander *et al*. [18] observed a type of small flat dysplastic lesions that was denoted as ACFMin in Min/+ mice, an Apc/familial adenomatous polyposis model. ACFMin exhibited dysplastic crypts similar to those found in adenomas and only those ACFMin showed a continuous development from the monocryptal stage to adenoma with rapid multiplication of crypts and altered expression of β-catenin. In contrast, the classic elevated ACF are hyperplastic, slow-growing, and show normal expression of β-catenin suggesting they are not directly associated with tumorigenesis [16-18].

The Wnt pathway controls the cell fate during the embryonic development. It not only drives tumorigenesis but also is required at different stages during the gut development. Activated Wnt signal pathway precludes the posttranslational down-regulation of β-catenin, which consequently accumulates in the cytoplasm and translocates into the nucleus where it complexes with the transcription factor Tcf-4 and activates specific target genes (such as c-MYC, cyclin D1 and MMP-7, etc.) [19].

In this study we evaluated the relationship between ACF and tumorigenesis by observing the sequential development from ACF to tumor in AOM-treated rats. In addition, we histochemically examined the expression of β-catenin and mmp-7.

## Methods

### Animals and chemicals

Sixty male SPF Wistar rats (8 to 9 weeks old; ~180 g weight) were provided by Nanfang Medical University animal center. the animal use is approved by the Animal Care and Use committee (approval number: 2002-009 2004B023 2004A068)and all the protocols are in agreement to the requirement of the committee. AOM (Sigma, St. Louis, MO, USA) was dissolved in 0.9% NaCl immediately before use and adjusted to pH 6.5–7.0. Rats were injected subcutaneously with azoxymethane (15 mg/kg bw/injection) once a week for 2 weeks. Animals were sacrificed sequentially from weeks 8 to 25 after the last injection of azoxymethane. The colons stained with methylene blue were transilluminated in the stereomicroscope, and the colonic lesions were characterized and scored based on the morphology of the tissues. The lesions were dissected and characterized by histopathological (for experimental details, see Table 1) and immunohistochemical analysis.

**Table 1:** Histopathologic examination of classic ACF, dark ACF, and tumor

	Hyperplasia without dysplasia	Mild dysplasia			Morderate dysplasia			Severe dysplasia		
		
		W8-14	W16-21	W22-25	W8-14	W16-21	W22-25	W8-14	W16-21	W22-25
Classic ACF	165	0	0	0	0	0	0	0	0	0
Dark ACF	0	1	0	0	2	7	2	0	4	6
tumor	0	0	0	0	0	0	3	0	1	6

Note: No. rats (n) used at each time point after AOM treatment: Wk8, 10, 12, 14, 16, 18, 20, 21,22, 23, 24, 25 = 5, respectively.

### Scoring of classic ACF, dark ACF, and tumors

The colons were removed, rinsed in ice-cold PBS, slit open longitudinally, and fixed flat between two wet (PBS) filter papers for 48 hours in 10% neutral buffered formalin followed by 10 seconds staining with 0.2% methylene blue (Chemical Ltd, Guangzhou, China) dissolved in the same formalin solution. Deeply stained crypts were examined by transillumination in stereomicroscopy after staining. Classic ACF were characterized by their obvious enlarged crypts, slight elevation above the surrounding mucosa thickened layer of epithelial cells, increased pericryptal space, and their light blue staining. Dark ACF were characterized by having dark blue staining, moderate enlarged or small crypts that are elevated from the surrounding epithelium and mild enlarged or small compressed crypts that are not elevated from the surrounding epithelium. Thus, darker blue staining and compressed pit pattern were used as the criteria for identification of dark ACF. The size of the lesions was scored by crypt multiplicity (AC/lesion). The crypt multiplicity of lesions was determined by transforming the diameter (mm) scored with an eyepiece to the crypt multiplicity by using the following empirical formula: c = 20 × d^2^. The lesions containing >32 aberrant crypts were defined as tumors.

### Histopathological analysis

Areas with mucosal lesions identified by examining the surface of the whole colon preparations in the stereomicroscopy were dissected and embedded in paraffin wax. After embedding, the tissue samples were sectioned in parallel with the mucosal surface. If the lesions have more than 32 aberrant crypts, then the tissues samples were sectioned in vertical with the mucosal surface. All sections were stained with H & E. Blind examination of the lesions from different levels of the crypts was performed by two pathologists.

Histopathological classification was based on the following criteria. Hyperplasia with no dysplasia was characterized as having slightly dilated crypts with normal epithelium. Mild dysplasia was characterized as having elongated, crowded and pseudostratified nuclei with well preserved polarity and normal or slightly reduced number of goblet cells. Moderate dysplasia was characterized as having elongated, more crowded and pseudostratified nuclei with well preserved polarity and more reduced number of goblet cells than in mild dysplasia. Severe dysplasia was characterized as having enlarged, round or ovoid nuclei with prominent nucleoli. The polarity of the nuclear in severe dysplasia is partially lost and the number of the goblet cells is significantly reduced or completely lost.

### Immunohistochemical analysis

Paraffin-embedded formalin-fixed sections were prepared, deparaffinized, and rehydrated in xylene, graded alcohol, and water. Demasking was performed in microwave oven for 12 minutes in Tris-EDTA solution. To determine the expression of β-catenin, sections were stained with monoclonal anti-β-catenin antibody (Wuhan Boster Ltd, Cat. No. BA0426, Wuhan, China) at the dilution of 1:200 and counterstained with hematoxylin using a SP kit. To determine the expression of MMP-7, sections were stained with monoclonal anti-MMP-7 antibody (Wuhan Boster Ltd, Cat. No. BA2110) at the dilution of 1:200 and counterstained with hematoxylin using a SP kit. β-catenin expression was evaluated separately for the cell membrane, cytoplasmic and nuclear compartments. If more than 70% of the membranes in the lesion cells were positively stained by the antibody, the membranous expression of β-catenin in the lesions was judged to be normal. If less than 70% of membranes in the lesion cells were positively stained, the membranous expression of β-catenin was judged to be reduced. On the other hand, if more than 10% of the cells were positive for the β-catenin staining in cytoplasm/nucleus, it was considered as cytoplasm/nucleus β-catenin expression because normal colonic mucosal cell cytoplasm/nucleus shows negative β-catenin expression. Thus, if more than 10% of the lesion cells showed elevated β-catenin labeling in the cytoplasm and/or distinct nuclear staining, the lesion was recorded as having cytoplasmic overexpression and/or nuclear accumulation of β-catenin. Reduced cell membrane expression or cytoplasmic overexpression/nuclear accumulation are recognized as altered expression. Cytoplasmic overexpression/nuclear accumulation are also called ectopic expression. For MMP-7, the criterion for positive expression was the presence of prominent cytoplasmic staining.

### Statistical analysis

*X*^2 ^test was used to calculate the statistical difference of proportions between groups. Student's *t *test was used to compare two groups and one-way ANOVA was used to compare ranks between multiple groups.

## Results

### Stereomicroscopy and histopathological analysis revealed two types of ACF:classic ACF and dark ACF

**Figure 1:**
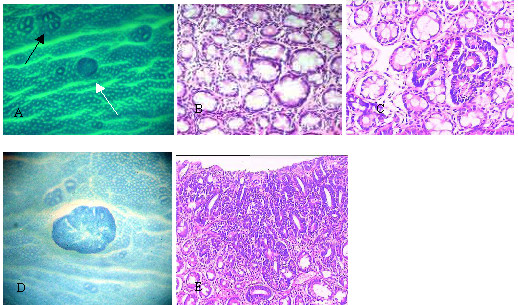
**Morphological observations of classic ACF, dark ACF and tumors**. Morphological observations of classic ACF, dark ACF and tumors. Lesions were stained with methylene blue and examined with the stereomicroscope (A, D, ×20); black arrowheads represent classic ACF and white arrowheads represent dark ACF. Histological observation (B, C, E) are derived from the same lesions as seen in A and D. Classic ACF showed hyperplasia without dysplasia (B, ×100); dark ACF showed moderate dysplasia (C, ×100); small tumor showed severe dysplasia (E, ×100).

Examination of the surface in the colon with stereomicroscopy revealed two types of ACF: classic ACF and dark ACF (Fig. 1A). Dark ACF were characterized by their darker blue staining than classic ACF. Furthermore, dark ACF have mild enlarged or small compact crypts that are not elevated from the surrounding epithelium. Histopathological examination showed that the lesions were classified into four categories: (1) hyperplasia without dysplasia; (2) mild dysplasia; (3) moderate dysplasia; and (4) severe dysplasia. All the classic ACF (165 cases) showed hyperplasia without dysplasia (Fig. 1B). The average crypt multiplicity for classic ACF slightly increased from 3.93 crypt/lesion at weeks 8~14 to 4.52 crypts/lesion at week 22~25 (F = 3.17, P = 0.278) (Table 2). In contrast, all the 22 dark ACF cases showed dysplasia with different degree (Fig. 1C) and the crypt with moderate or severe dysplasia accounted for 95.4% (21/22) of the cases. The average focal crypt multiplicity of dark ACF increased significantly between different categories (F = 15.4, P = 0.000), In addition, crypt multiplicity of dark ACF increased significantly faster than those of classic ACF.

**Table 2:** Crypt multiplicity of classic ACF, dark ACF, and tumors (mean ± SD)

	crypt multiplicity (No. of crypts/lesion/group)				
		
	W8-14	W16-21	W22-25	F	P
Classic ACF	3.93 ± 0.89	4.73 ± 1.12	4.52 ± 1.03	3.17	0.278
Dark ACF	6.00 ± 3.00	10.73 ± 4.03	18.38 ± 3.54	15.40	0.000
t	3.330	9.883	11.204		
P	0.002	0.000	0.000		

### Dark ACF is phenotypically similar to tumor

Nascent tumors exhibited characteristic of dark blue staining and contained branched or gyrus-like pit pattern of compressed crypts (Fig. 1D). In addition, nascent tumors displayed the same surface morphology as dark ACF. All the tumors showed moderate or severe dysplasia (10 of 10). There was no significant difference between the proportion of morderate/severe dysplasia in dark ACF (95.4%) and that in tumors (100%). The rapid increase of crypt multiplicity in dark ACF is similar to the characteristic of tumors.

### Expression of β-catenin in classic ACF, dark ACF and tumors

**Figure 2:**
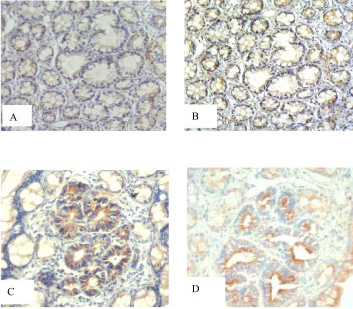
**Immunohistochemical analyses for the expression of β-catenin and mmp-7 in classic ACF, dark ACF and histological sections of lesions**. Immunohistochemical analyses for the expression of β-catenin and mmp-7 in classic ACF, dark ACF and histological sections of lesions. The expression of β-catenin (A) and mmp-7 (B) is not altered in classic ACF. The expression of β-catenin (C) and mmp-7 (D) is altered in dark ACF. Magnification: ×100.

The immnoreactivity of β-catenin in the classic ACF, dark ACF and tumors is summarized in Table 3. Expression of β-catenin is normal in most of the classic ACF (157 of 165, Fig. 2A). Four of the classic ACF showed reduced expression in cell membrane and the other four classic ACF showed cytoplasmic expression of β-catenin. In contrast, 72.72% (16/22) of dark ACF showed cytoplasmic overexpression or nuclear accumulation (Fig. 2C). All the ten tumor cases showed cytoplasmic overexpression or nuclear accumulation of β-catenin. Interestingly, the expression pattern of β-catenin for dark ACF or tumors was mainly characterized by reduced expression in membrane during the early period (week 8~14) and then transited to nuclear accumulation during the later period (week 22~25) (Table 4).

**Table 3:** Immunohistochemical analysis for the expression of β-catenin, mmp-7 in classic ACF, dark ACF and tumor

	β-catenin				mmp-7	
	No. of normal samples/total No. of samples	No. of samples with reduced expression in membrane/total No. of samples	No. of samples with overexpression in cytoplasm/total No. of samples	No. of samples with accumulated expression in nucleus/total No. of samples	No. of samples with mmp-7 expression/total No. of samples	No. of samples without mmp-7 expression/total No. of samples

Classic ACF	157/165	4/165	4/165	0/165	13/165	152/165
Dark ACF	0/22	6/22	8/22	8/22	18/22	4/22
Tumor	0/10	0/10	3/10	7/10	10/10	0/10

Note: The rate of ectopic expression of β-catenin in classic ACF is significantly lower than that in tumors (*P *= 0.0023). The rate of mmp-7 expression in classic ACF is significantly lower than that in tumors (*P *= 0.000). The rate of β-catenin ectopic expression and positive expression of mmp-7 in dark ACF is not significantly different with those in tumor (*P *= 0.143 and *P *= 0.283, respectively). The rate of ectopic expression of β-catenin in dark ACF is significantly higher than that in classic ACF (*P *= 0.000). The rate of mmp-7 expression in dark ACF is significantly higher than that in classic ACF (*P *= 0.000).

**Table 4:** Expression of β-catenin in dark ACF at different time point

	β-catenin expression		
	No. of samples with reduced expression in membrane/total No. of samples	No. of samples with overexpression in cytoplasm/total No. of samples	No. of samples with accumulated expression in nucleus/total No. of samples

Wk8-14	3/3	0/3	0/3
Wk16-21	3/11	5/11	3/11
Wk22-25	0/8	3/8	5/8

### Expression of mmp-7 in classic ACF, dark ACF and tumors

The immnoreactivity of mmp-7 in the classic ACF, dark ACF and tumors is summarized in Table 3. The expression rate of mmp-7 in dark ACF (Fig. 2D) was 81.82% (18/22), which was significantly higher than that in classic ACF (7.87%, 13/165) (Fig. 2B). All the tumor cases exhibited cytoplasmic overexpression of mmp-7. Furthermore, there was no significant difference between expression of mmp-7 in dark ACF and tumors.

## Discussion

By examining the sequential development of early lesions in the colon of Wistar rats 8 to 25 weeks after AOM treatment, we identified two types of lesions: classic ACF and dark ACF. Dark ACF were characterized by their darker blue staining than the classic ACF. In addition, dark ACF has mildly enlarged or small crypts that are not elevated from the surrounding epithelium. The crypts of dark ACF is compact and similar to a compressed luminal opening. Dark ACF shares similarities with tumors with regard to the surface morphology of the colon, higher crypt multiplicity, pathologic classification and expression pattern of β-catenin and mmp-7. However, classic ACF are different to tumors. These results may explain why only a few colon tumors are observed although hundreds of ACF are induced per animal. Activation of β-catenin, a core protein in Wnt signal transduction pathway in nucleus, correlates with the tumorigenesis and abnormal accumulation of β-catenin plays an important role in the development of tumors [20-22]. One of the studies found that β-catenin is less expressed in the membrane of ACF, but more expressed in cytoplasm and nucleus when ACF variation increases [23]. Our study showed that the ectopic expression ratio of β-catenin was 72.7% in dark ACF, which had no significant difference compared with that in tumors. In addition, expression pattern of β-catenin transited from reduced cell membrane expression in the early period to nuclear accumulation in the later period. Simultaneously, activation of oncogene encoding β-catenin could stimulate the downstream target, MMP-7, to suppress tumor cell apoptosis and degradation of basic membrane. Duan *et al*. [24] found that expression of β-catenin in cytoplasm and nucleus directly correlated with the expression of MMP-7 protein, suggesting that abnormal expression of β-catenin could initiate Wnt signal transduction pathway leading to the expression of MMP-7. Our study found that β-catenin ectopic expression ratio was positively correlated with the MMP-7 positive ratio, and meanwhile, there was no statistical difference between the expression of MMP-7 in dark ACF and that in tumors. These results suggested that increased β-catenin accumulation in dark ACF activated the Wnt pathway leading to the loss of cell adhesion and separation. Loss of cell adhesion and separation resulted in more contact between cells and high expression of MMP-7, leading to further degradation of the outer membrane and unlimited cell growth. Dark ACF obtained the ability to grow invasively and was converted to colon cancer. We found that β-catenin and MMP-7 expression was not changed in classic ACF, which suggested that oncogene activation and expression does not exist in classic ACF. Therefore, the chance of conversion from classic ACF to tumors is relatively small.

## Conclusion

In summary, we identified two types of ACF: dark ACF and classic ACF. Dark ACF is similar to tumor with regard to its morphological and pathological changes, the increased number of crypts and the expression oncogenes. However, all these features identified in dark ACF and tumors were not observed in classic ACF. These results suggested that not all ACF are early indications of colon cancers and only a specific type of ACF (dark ACF) may be used for diagnosis and evaluation of anticancer abilities of chemicals and food additives. Furthermore, our results also showed that the key proteins in the Wnt signal transduction pathway were abnormally expressed suggesting that the activation of Wnt pathway exists in the stage of ACF during the cancer development. Future studies will be focused on the pathological changes and activation of signal transduction pathways during earlier stages of ACF.

## Abbreviations

ACF: aberrant crypt foci; AOM: azoxymethane.

## Authors' contributions

LQ: Principal investigator and Experimental Designer. JB: Department head and Experimental Designer. CL, TS: Technician.
